# Developing a Measure to Quantify Ocular Pain Postoperatively: The Adaptation of the Ocular Pain Assessment Survey

**DOI:** 10.1155/2022/3116913

**Published:** 2022-10-14

**Authors:** Ayse Yildiz-Tas, Sadi Can Sonmez, Zeynep Busra Kisakurek, Gulsum Deniz, Arzu Baygül, Cem Kesim, Melisa Zisan Karslioglu, Cem Ozturkmen, Rengin Aslihan Kurt, Pedram Hamrah, Afsun Sahin

**Affiliations:** ^1^Koç University Hospital, Department of Ophthalmology, Istanbul, Turkey; ^2^Koç University School of Medicine, Istanbul, Turkey; ^3^Reserach Center for Translational Medicine, Koç University, Istanbul, Turkey; ^4^Department of Biostatistics and Medical Informatics, Koç University, Istanbul, Turkey; ^5^Gaziantep Goznuru Eye Hospital, Gaziantep, Turkey; ^6^Baskent University Hospital, Department of Ophthalmology, Istanbul, Turkey; ^7^Center for Translational Ocular Immunology, Department of Ophthalmology, Tufts Medical Center, Tufts University School of Medicine, Boston, MA, USA; ^8^Tufts Medical Center, Tufts University School of Medicine, Boston, MA, USA; ^9^Cornea Service, New England Eye Center, Department of Ophthalmology, Tufts Medical Center, Tufts University School of Medicine, Boston, MA, USA

## Abstract

**Purpose:**

Since quantification and communication of ocular pain is important for a healthier patient follow-up and postoperative guidance, reliable measures like the Ophthalmic Pain Assessment Survey (OPAS) are needed to assess the outcome and management of different operations. To address that need, we carried out the adaptation of OPAS into Turkish to reach different age groups and backgrounds, widening the use of OPAS on patients who underwent an ophthalmic operation.

**Methods:**

We used back-translation method and achieved cultural adaptation through content validity scoring by 5 independent ophthalmologists. The survey is then administered three times: preoperatively, postoperatively within 24 hours, and finally a week later in the follow-up visit. Validity is measured in comparison to Visual Analog Scale using Spearman's correlation coefficient and reliability is measured using Cronbach's alpha. Factor analysis is performed by principal component analysis and rotation is performed using Varimax method when necessary.

**Results:**

We reached a total of 132 patients with a mean age of 64.2 years. Most of them underwent phacoemulsification (*n* = 83), followed by PRK (*n* = 37). Overall, the T-OPAS demonstrated good reliability (mean C. alpha: 0.830) and its correlation with the VAS was especially high (S. coeff. >0.5) in the first three sections in all three surveys. Factor analysis yielded 5 subscales, allowing us to shape the final form of T-OPAS.

**Conclusion:**

Through this adaptation of OPAS into a foreign language, we present a reliable and valid tool for postoperative pain quantification, allowing objective measurement of pain in different populations such as the elderly.

## 1. Introduction

Ocular pain is an important finding in ophthalmology that can have both orbital and extra-orbital causes [1, 2]. It can manifest itself in various presentations including photophobia, itching, or discomfort with movement. The differential diagnosis of ocular pain is also extensive and requires the perspective of both ophthalmology and neurology [2, 3]. Among the various causes, conjunctivitis, blepharitis, chalazion, corneal abrasion, and dry eye disease are the most common ones [1, 2].

Several ophthalmic procedures are also known to cause ocular pain, mostly for a transient duration of time in the postoperative period. The type of the procedure, along with many other factors, influences the pain intensity and the associated symptoms such as light sensitivity (photophobia), foreign body sensation, burning, tearing, and itching. For instance, in refractive surgery (especially PRK), a very common intervention that involves laser treatment to the cornea, a considerable postoperative pain due to the stimulation of the corneal nerve endings is expected [4]. The pain peaks around twenty-four hours after the surgery and can be very severe, reportedly around eight on a ten-point scale [4]. It is also associated with photophobia, tearing, and burning sensation [4, 5]. This is different from what is generally expected after cataracts surgery thanks to new techniques such as micro-incisions and cold irrigation of the eye, intraoperatively [6, 7]. Unlike laser eye surgeries, in cataract surgery, patients report discomfort to some degree along mostly with foreign body sensation, but they report a lesser degree of photophobia [8]. However, pain with an unusual duration and intensity can signal endophthalmitis and increased intraocular pressure due to inflammation [9–11]. As another example, in trabeculectomy, less pain is generally expected and any ocular discomfort should be followed well and correlated with the intraocular pressure levels to detect complications earlier [12]. Aside from the anterior chamber procedures, pars plana vitrectomy which includes the manipulation of the vitreous chamber is also associated with pain and foreign body sensation even a month after the surgery [13]. Some patients can continue to report irritation as in foreign body sensation and dryness months later after the surgery even if the pain subsides [13]. Irrespective of the procedure itself, untreated dry eye disease, preoperatively elevated intraocular pressure, and concomitant systemic diseases can also be independent factors affecting pain [9–12].

Ophthalmologists therefore need to be vigilant about pain and related symptoms before, during, and after a procedure in order to detect and treat complications [14–16]. Ophthalmic surgeons should monitor the change in the character/intensity of pain as it can be an important red flag for various conditions [6, 12, 14–16]. They should especially inquire postoperative pain in certain populations that are prone to complications such as the elderly or patients with concomitant vascular diseases [17]. That is why it is important to develop measures which try to quantify pain in each individual patient for orbital surgeries. With this regard, it is also significant to establish good communication with the patients to guide them in the postoperative process especially after procedures where considerable pain is expected such as the PRK. This can be helpful to differentiate what is unusual from what is expected, faster.

The Ophthalmic Pain Assessment Survey (OPAS) has been proven to be a reliable and valid inventory to assess multidimensional orbital and extra-orbital pain [18]. It helps to objectify pain using subjective scores given by the patient and provides easy follow-up for the course of pain. It is also helpful to distinguish ophthalmic and nonophthalmic pain and allows better differential diagnosis. The survey analyzes pain in categories such as quality of life (QoL), exacerbating factors, and change over duration. This allows better understanding of the patient's situation and can direct the physician about the most suitable pain management option [19]. We believe that the use of such a survey in postoperative patients can also be beneficial in quantifying and categorizing the ocular pain. It can also be beneficial during the COVID19 pandemic since the elderly patients hesitate to visit the hospital and certain symptoms can remain unnoticed until it is too late [3]. In that sense, we believe that even a remote application of such a survey with regular intervals can improve the current state of care in ophthalmology today.

In Turkey, there is no standardized measure of pain in the ophthalmology practice. We believe that this is problematic in the daily practice since there are no means to quantify and compare the level of pain preoperatively and postoperatively for the same patient. There is also a lack of reporting on how this situation affects unnecessary presentations to the clinic or on the other hand, if there is any delay in the diagnosis of complications. In Turkey, some subspecialties have adapted international pain scales and used them in their daily practice successfully [20–23]. Similar studies are also present in other countries about adaptations of different scales into their own practice [24–26]. We, therefore, find it necessary to create an adaptation based on OPAS in accordance with Turkish language and cultural components to adopt a standardized system of reporting pain in ophthalmology. We believe that this adaptation can prove OPAS to be a reliable tool not only in acute conditions but also in preoperative and postoperative pain assessment.

## 2. Materials and Methods

### 2.1. Study Design

The original Ocular Pain Assessment Survey is considered a gold standard in eye pain evaluation and consists of 6 sections and 24 questions in total [18, 19]. In the first two sections, eye pain is quantified according to duration—last 24 hours and last 2 weeks—and severity. In the third section, noneye pain is quantified in a similar fashion. In the last three sections, quality of life (how much it affects certain activities like reading or driving), aggravating factors (i.e., wind, dry air, and heat), and associated factors (i.e., redness, burning) are asked and any improvement since the last visit are noted. Each section and question are scored independently (from 0 to 10) and compared with previous records of the patients. To conduct this study, permission from the first author (Hamrah et al.) of the original OPAS was obtained via e-mail.

The adaptation process began by the translation of the survey into Turkish, following a back-translation method as established by similar validation studies [20–26]. The forward translation was carried out by two independent ophthalmologists and then reviewed by the team members for comprehension and transcultural consistency. Then, it was reverse translated into English by two different native English-speaking researchers to compare with the original. To evaluate content validity, five independent ophthalmologists scored all items from 0 to 5. Items that scored lower than 4 were modified by the team until a consensus is reached and a higher score is assigned. The final product is constructed when all elements received a mean score no less than 4 after modifications. It is accessible as Digital Supplementary Content 1.

In order to check for certain parameters of the survey, a cross-sectional scheme is constructed. The study included adult patients who underwent surgery after visiting the clinic from November 2020 to April 2021. All the participants were volunteers who provided formal consent upon written and verbal explanation prior to their surgery date. The patients who could not communicate in Turkish, had an extra-ocular procedure simultaneously or not long ago, had general anesthesia or did not show up to their follow-up visits have been excluded from this study.

**Figure 1:**
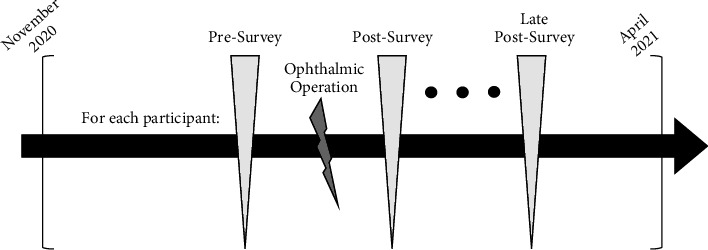
The timeline of our study design. Note that each survey is the same version of the adapted OPAS in Turkish, only difference being the time it was administered. The same scheme was repeated for each of the 60 patients. The control visit in which the late-postsurvey took place in the next day of surgery.

We planned to apply the same survey three times at different time-points as presurvey, postsurvey, and late-postsurvey (Figure 1) for pain.

The presurvey was administered to the patients 10 minutes before their planned surgery, whereas the postsurvey was administered the next morning during the first follow-up visit to cover the first postop for 24 hours. Late-postsurvey was administered a week later during the second follow-up visit. The last section that quantified improvement in the patient's pain is asked only in the late postsurvey since pain level was expected to rise postop.

### 2.2. Statistical Analysis

In our study, the responses given to quantify pain were considered as a continuous variable from 0 to 10, from which we calculated the mean, standard deviation, minimum, maximum, and median values. In addition to that, the frequency and percentage values are calculated to identify other categorical variables our survey measured such as the type of the surgery and the demographics of the patients. We used the Kaiser–Meyer–Olkin test to calculate the adequacy of our sample size. As generally accepted, a value between 0.8 and 1 indicated a good sample size.

In order to develop an applicable survey that elicits consistent responses which correspond well with the pain and associated symptoms, we tested our adaptation's validity and reliability using established statistical methods [20–26].

To determine the validity of our survey and how well it measures pain, we calculated the correlation of our adaptation with the gold standard Visual Analog Scale (administered alongside the survey) by using Spearman's Rho coefficient. A higher correlation value indicated a relatively more successful measure of pain. We further analyzed the variability within our survey by using factor analysis with varimax rotation method, as in previous studies [20, 26]. This method allowed us to detect statistical correlations within our survey and further guided us on constructing our adaptation and its sections.

We measured the reliability of the items within the survey and how well the items were consistent with one another using the Cronbach's alpha coefficient, as in previous studies [20–26]. A value greater than 0.7 indicated good internal consistency and therefore the result of the question within the survey was reliable.

Statistical analysis of the collected data is performed using IBM SPSS statistics (Version 26.0. Armonk, NY) for Windows. Level of statistical significance was determined as *p* < 0.05.

## 3. Results

### 3.1. Demographics

The total number of patients included in the study was 132 with a mean age of 64.2. The patients underwent operations including phacoemulsification (*n* = 83), PRK (*n* = 37), LASIK (*n* = 5), pterygium excision (*n* = 2), eyelid surgery (*n* = 2), intravitreal injection (*n* = 2), and trabeculectomy (*n* = 1). 29 of the refractive surgery patients underwent bilateral surgery; however, the rest of the patients had unilateral surgery (nOD = 55, nOS = 48). 44 of the patients (*n* = 44, 33.3%) reported additional nonophthalmic pain prior to the surgery with headache being the most common (*n* = 31, 23.5%) and neck pain being the second most common (*n* = 10, 7.6%).

### 3.2. Characteristics of Change in Pain Severity

The answers given to questions in the three surveys are summarized numerically (Table 1).

The increase in the mean of reported pain and symptoms from presurvey to postsurvey is evident in most sections with statistical significance. We could observe an abating trend in pain and associated symptoms as expected in the late-postsurvey especially in section 3, but statistical significance was not achieved in each item.

**Figure 2:**
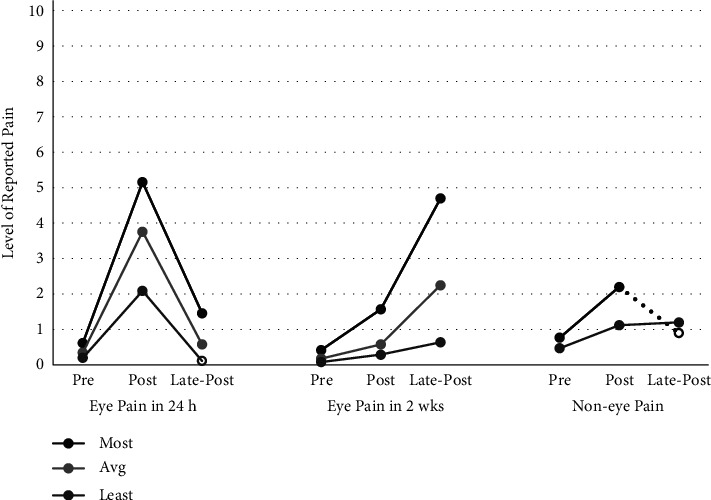
The change in the level of reported pain for certain parameters. Mean values are used to construct the lines. Significant changes from pre- to postsurvey and from postsurvey to late-postsurvey are shown as full lines. Significant changes from presurvey to late-postsurvey are shown as full points at the end of each graph. Note that no average pain was questioned for noneye pain and therefore it is not present in the figure.

The change in the level of pain is visualized as bar charts of certain items (Figure 2).

As seen, daily pain (eye pain in 24 h) increased significantly (*p* < 0.001 for all three of the scales) within the postoperative 24 hours. It then decreased to preop values in the late-postsurvey within 1 week after surgery (*p* < 0.001 for all three of the scales). This affected the 2-week pain levels as an increase in the past level of pain. Noneye pain also increased postop significantly (*p* < 0.001 for all three of the scales). No statistically significant difference between the characteristics of the change in pain levels could be shown between the phacoemulsification and refractive surgery patients and therefore it is not shown here.

### 3.3. Reliability

The reliability analysis overall yielded sound results (Table 2).

All scores were higher than 0.700 except subscale 4 of pre and late-postsurveys and subscale 5 of late-postsurvey. The highest internal consistency was achieved in the presurvey with Cronbach's alpha scores of almost 1.

### 3.4. Validity

We could achieve varying levels of correlation of each item with the universal gold standard Visual Analog Scale (VAS) (Table 3).

Stronger correlations are observed in the first two sections asking directly about the eye pain over a period of 24 hours and 2 weeks. Correlations fall weaker in the last two sections asking about aggravating and associated factors. Especially in the late-postsurvey, aggravating factors were not statistically correlated with the gold standard measure of pain. Following that, noneye pain section in the postsurvey was also faintly correlated with VAS.

### 3.5. Factor Analysis

Kaiser–Meyer–Olkin test to determine sample adequacy yielded a value of 0.851, indicating a good sample size. Following that, factor analysis was performed separately for the presurvey, postsurvey, and late-postsurvey. Postsurvey results are further individualized based on the type of the surgery, focusing especially on cataract patients who underwent phacoemulsification and patients of laser surgery. Among all these analyses, total postsurvey results had the best rotated component distribution and therefore chosen as the main guide for the sectioning of our adaptation (Table 4). Cumulative variance was 75.87% for 5 subscales in total. The final form of the Turkish OPAS is therefore structured and categorized accordingly.

## 4. Discussion

Our study revealed that the Turkish version of OPAS is a reliable and valid questionnaire to assess ocular pain and associated symptoms, widening the use of OPAS as a valid tool to remotely assess surgical pain of the orbit. Using this survey, ophthalmic pain can now be better expressed and followed easily by the physicians in the postoperative period and the general practice. It can also be used to compare the pain characteristics after different operations internationally and among different cultures, backgrounds, age groups, and methods.

The patient population that we have included in this study was mostly in the 6th decade of life which is known to constitute a barrier on its own in verbal pain expression [27, 28]. We believe that the Turkish OPAS can guide the physician when communicating with this population to get a clearer view of the symptoms. It is also inclusive in the means of the range of symptoms that exhibit itself as pain since pain in Turkish has multiple connotations [29]. Depending on the background of the person and the situation, a patient can report inadequate pain as he or she might be exhibiting only a single connotation of pain. For example, the patient can report to have moderate pain while having excessive burning or aching in the eye since they are different connotations of the word pain in Turkish and it is hard for the elderly to describe each. By questioning symptoms separately and using broader terms to describe pain, the Turkish version of OPAS can be helpful in preventing misunderstandings and enhance the access to right care. Further studies, therefore, on the use of OPAS as an assessment tool should always acknowledge such possible nuances based on the target population and region.

The reliability of the survey was also found high as almost all items were between 0.7 and 1 (Table 2). Especially subcategory 4 (exacerbating factors such as wind or fumes) in the presurvey and late-post survey had the lowest Cronbach values. This might be partially due to patients becoming overprotective of their eyes before and after the surgery and avoiding any potential volatile substance. The lowest reliability score however is observed in the late-postsurvey in subcategory 5 (Associated Factors). We realized this was observed because most redness and burning resolved within the first postop week in this patient group despite some residual pain. Therefore, the timing of the survey can interfere with the reliability of this subcategory.

Correlation levels with the gold standard pain measure of VAS were measured the highest in the first subcategories and declined towards the subcategories 4 and 5, generally speaking (Table 3). In addition to the same factors that influenced the reliability of these subcategories, temporary closure (12 hrs postop) of the eyes of the phacoemulsification surgery patients (a majority of our patient population) could have also decreased the correlation of associated and exacerbating factors with pain. Low levels of correlation with the 2-week pain levels in the postsurveys were expected since the pain elicited by the surgery was excessive compared to the levels of pain felt in the past weeks. The similar was also expected for the late-post survey since the pain felt due to the surgery in the past week had already elapsed by the time the late-postsurvey was filled out. Another important thing to note can be the decrease of correlation in the noneye pain section when pre- and postsurveys are compared. This is probably because the patient ignored his or her regular aches and sores for a short amount of time after the ocular surgery.

The KMO test revealed excellent results with regard to our sample size and the principal component analysis yielded 5 main subscales of our survey (Table 4). The main reason why we selected the postsurvey results instead of the general results was because the surgery was the main painful event and therefore measuring pain postoperatively as close to the event as possible would understandably reveal the best analysis. Having said that, the only difference in the subscales from the original OPAS was observed in the “Quality of Life” and “Associated Symptoms” sections. As suggested by these statistics, we have decided to merge these two sections into one and refer to it as the “Additional Symptoms and Effects” in the final form of the survey (Digital Supplementary Content 1).

We have certain limitations in this study. First of all, managing an elderly population—presenting usually with multiple co-morbidities—was especially challenging during the COVID19 pandemic. This also affected our patient diversity as elective operations were not preferred by most ophthalmology patients during this period. Secondarily, we did not consider any difference in education level, cultural background, socioeconomics, and certain other painful comorbidities like arthralgia which might have affected the patient's perception of pain. Physicians might need to take these into account when they administer this test in their practice or when they compare the results of different patient populations. Another limitation was the difference between the age groups undergoing a cataract surgery and the ones—who were younger—undergoing laser surgeries. Although no significant difference was seen between these two groups, their different pain thresholds might have influenced their answers [30].

## 5. Conclusions

With this study, we present a reliable and valid Turkish form of the original OPAS developed by Hamrah et al. [18, 19] which is also the first adaptation to widen the survey's use in another language and patient population. Our adaptation has demonstrated a good level of statistical validity and reliability and therefore can be deemed applicable to be used in the daily practice. We hope that this will in turn help the clinic and the patients to follow their symptoms despite the current barriers due to the COVID19 pandemic.

Additionally, we believe this survey and its possible adaptations into other languages will be helpful in establishing a better line of communication with the clinic and the patient in the postoperative period. We aim that it helps with an easier recovery process and quicker detection of complications and secondary infections in especially risk-bearing populations.

Finally, with its different sections, the survey targets multiple aspects of the postoperative period and therefore can be useful in providing a holistic summary of the patient's wellbeing. As pain is also a multidimensional concept with lots of associated symptoms in ophthalmology, we believe that such a tool can make it easier for the physicians to track their patient's overall experiences.

## Figures and Tables

**Table 1:** The statistics of the answers given to pre, post, and late-postsurveys. Each section is evaluated from 0 to 10. The final section on pain relief is not included since it is only tested in the late-postsurvey. Overall significance is measured statistically via the Friedman test, whereas individual significances are calculated using Wilcoxon signed rank test. P1 is the significance between presurvey and postsurvey where P2 is the significance between post and late-postsurveys and P3 is the significance between pre and late-postsurvey. Bonferroni adjustment is made to determine the significance of the three surveys (significance <0.05/3).

Item no.	PRE		POST		LATE-POST		(*P*-Values)			
	Mean + std	Median (min-max)	Mean + Std	Median (min-max)	Mean + Std	Median (min-max)	Overall significance	*P*1	*P*2	*P*3
1.1	0.62 + 1.58	0 (0–10)	5.16 + 2.26	5 (0–10)	1.46 + 1.19	1 (0–4)	<0.001	<0.001	<0.001	<0.001
1.2	0.20 + 0.84	0 (0–6)	2.09 + 1.85	2 (0–9)	0.11 + 0.40	0 (0–3)	<0.001	<0.001	<0.001	0.434
1.3	0.35 + 1.05	0 (0–5)	3.76 + 2.00	4 (0–10)	0.58 + 0.84	0 (0–3)	<0.001	<0.001	<0.001	0.001
1.4	0.42 + 1.25	0 (0–9)	1.57 + 1.60	1 (0–7)	4.70 + 2.05	5 (0–10)	<0.001	<0.001	<0.001	<0.001
1.5	0.08 + 0.59	0 (0–6)	0.29 + 0.90	0 (0–6)	0.64 + 0.89	0 (0–5)	<0.001	<0.001	<0.001	<0.001
1.6	0.18 + 0.73	0 (0–6)	0.58 + 1.00	0 (0–6)	2.25 + 1.29	2 (0–6)	<0.001	<0.001	<0.001	<0.001
2.1	0.77 + 1.44	0 (0–9)	2.20 + 2.30	2 (0–9)	0.90 + 1.32	0 (0–6)	<0.001	<0.001	<0.001	0.075
2.2	0.47 + 0.97	0 (0–5)	1.12 + 1.71	0 (0–9)	1.20 + 1.42	1 (0–5)	<0.001	<0.001	0.319	<0.001
2.3	0.38 + 1.02	0 (0–7)	1.32 + 1.95	0 (0–10)	0.45 + 0.96	0 (0–5)	<0.001	<0.001	<0.001	0.375
3.1	0.92 + 1.90	0 (0–9)	4.32 + 2.20	4 (0–10)	1.36 + 1.31	1 (0–6)	<0.001	<0.001	<0.001	<0.001
3.2	0.49 + 1.25	0 (0–6)	3.84 + 2.48	4 (0–10)	0.77 + 1.00	0 (0–5)	<0.001	<0.001	<0.001	<0.001
3.3	0.41 + 1.00	0 (0–6)	3.55 + 2.31	3 (0–10)	0.81 + 0.81	1 (0–3)	<0.001	<0.001	<0.001	<0.001
3.4	0.21 + 0.76	0 (0–6)	2.82 + 2.29	2 (0–10)	0.36 + 0.70	0 (0–3)	<0.001	<0.001	<0.001	0.015
3.5	0.21 + 0.74	0 (0–4)	1.71 + 2.10	1 (0–10)	0.15 + 0.50	0 (0–3)	<0.001	<0.001	<0.001	0.362
3.6	0.28 + 0.85	0 (0–6)	3.08 + 2.30	2.5 (0–10)	0.45 + 0.69	0 (0–3)	<0.001	<0.001	<0.001	0.002
3.7	0.31 + 0.96	0 (0–7)	3.52 + 2.39	3 (0–10)	0.55 + 0.82	0 (0–4)	<0.001	<0.001	<0.001	<0.001
4.1	0.75 + 1.62	0 (0–8)	2.66 + 2.37	2 (0–9)	1.19 + 1.38	1 (0–7)	<0.001	<0.001	<0.001	<0.001
4.2	0.30 + 1.03	0 (0–6)	2.18 + 2.47	1 (0–8)	0.55 + 0.88	0 (0–4)	<0.001	<0.001	<0.001	0.002
5.1	0.07 + 0.50	0 (0–5)	3.13 + 3.22	2 (0–10)	0.17 + 0.52	0 (0–3)	<0.001	<0.001	<0.001	0.017
5.2	0.37 + 1.33	0 (0–10)	4.36 + 2.70	4 (0–10)	0.94 + 0.98	1 (0–5)	<0.001	<0.001	<0.001	<0.001
5.3	0.44 + 1.47	0 (0–10)	3.83 + 3.17	4 (0–10)	0.96 + 1.06	1 (0–6)	<0.001	<0.001	<0.001	<0.001
5.4	0.64 + 1.47	0 (0–6)	3.08 + 3.14	2 (0–10)	0.58 + 1.04	0 (0–5)	<0.001	<0.001	<0.001	0.620

**Table 2:** The Cronbach's alpha values of each subscale. The final section on pain relief is not included since it is only tested in the late-postsurvey.

Subcategories	Cronbach's Alpha Value			
	No. of items	Presurvey	Postsurvey	Late-postsurvey
Overall	22	0.948	0.931	0.900
1. Eye pain severity	6	0.925	0.789	0.734
2. Noneye pain severity	3	0.906	0.872	0.855
3. Quality of life	7	0.910	0.942	0.852
4. Exacerbating factors	2	0.624	0.816	0.640
5. Associated factors	4	0.788	0.895	0.623

**Table 3:** The Correlation between the gold standard VAS pain measurement and the subscales of Turkish adaptation of OPAS. The Spearman's correlation coefficient is calculated for each item in the survey. The final section on pain relief is not included in the analysis. A strong correlation is defined with a correlation coefficient of approximately 0.6, whereas a low correlation is defined with a coefficient between 0.3 and 0.6. Significance is determined as <0.05.

Items		PRE		POST		LATE-POST	
		Correlation coefficient	Significance (*P*-value)	Correlation coefficient	Significance (*P*-value)	Correlation coefficient	Significance (*P*-value)
24 h eye pain	Most	0.677	<0.001	0.789	<0.001	0.623	<0.001
	Least	0.467	<0.001	0.478	<0.001	0.155	0.076
	Avg.	0.660	˂0.001	0.741	<0.001	0.589	<0.001

2 wks eye pain	Most	0.556	˂0.001	0.272	0.002	0.247	0.004
	Least	0.269	˂0.001	0.258	0.003	0.094	0.288
	Avg.	0.555	˂0.001	0.094	0.283	0.244	0.005

Noneye pain	Most	0.577	<0.001	0.202	0.020	0.371	<0.001
	Least	0.542	<0.001	0.156	0.075	0.305	<0.001
	Avg.	0.452	<0.001	0.198	0.023	0.400	<0.001

Quality of life	Reading/Computer	0.499	<0.001	0.483	<0.001	0.327	<0.001
	Driving/TV	0.565	<0.001	0.518	<0.001	0.362	<0.001
	General	0.544	<0.001	0.547	<0.001	0.354	<0.001
	Mood	0.402	<0.001	0.526	<0.001	0.289	0.001
	Sleep	0.542	<0.001	0.416	<0.001	0.261	0.003
	Relations	0.452	<0.001	0.373	<0.001	0.202	0.021
	Thinking abt. pain	0.592	<0.001	0.511	<0.001	0.464	<0.001

Aggravating factors	Wind/AC/Dry air	0.397	<0.001	0.332	<0.001	0.136	0.122
	Volatile chemicals	0.233	0.007	0.239	0.006	0.090	0.308

Associated factors	Red	0.174	0.046	0.210	0.016	0.276	0.001
	Burn	0.430	<0.001	0.400	<0.001	0.288	0.001
	Photosensitivity	0.422	<0.001	0.253	0.003	0.198	0.023
	Tearing	0.350	<0.001	0.222	0.010	0.102	0.247

**Table 4:** The rotated component matrix generated by principal component analysis (using varimax rotation) of our survey items. The 5 subscales generated by the analysis are colored with the same shade. The final section on pain relief is not included in the analysis.

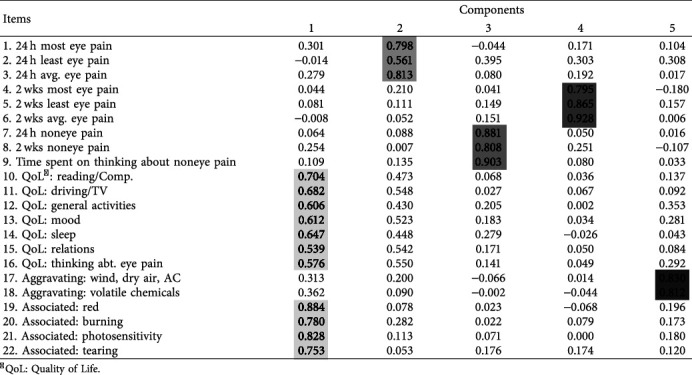

## Data Availability

The data used to support the findings of this study are available upon request through corresponding author.
