# Supplementary material for: Developing a Measure to Quantify Ocular Pain Postoperatively: The Adaptation of the Ocular Pain Assessment Survey
Source: J Ophthalmol. 2022 Oct 14;2022:3116913. doi: 10.1155/2022/3116913 (PMC9586810; doi:10.1155/2022/3116913)

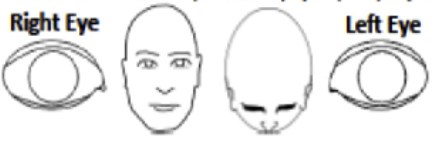

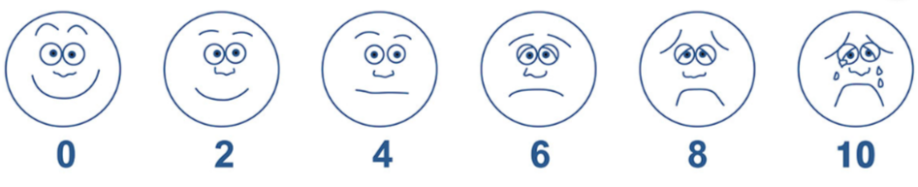
 Lüften bu formu yüzünüzde veya gözlerinizde ağrınız varsa doldurunuz.

Eğer cevabınız evet ise lütfen nerenizin ağrıdığını açıklayınız:________________

3. Vücudunuzun herhangi bir bölgesinde uzun süredir mevcut olan bir ağrı var mı? HAYIR

2. Aşağıda verilen şablonda, lütfen gözünüzde ve/veya yüz ve kafa bölgenizde ağrı duyduğunuz yerleri işaretleyiniz.

(VAS): Altın Standart Ağrı Ölçüsü

Hasta Bilgisi Etiketi

Hasta imzası:__________________ Tarih:__________________

**AŞAĞIDAKİ SORULARI LÜTFEN DAHA ÇOK AĞRIYAN GÖZÜNÜZE GÖRE CEVAPLANDIRINIZ**

Lütfen göz ağrınızın şiddetini aşağıdakilere göre yuvarlak içine alınız:
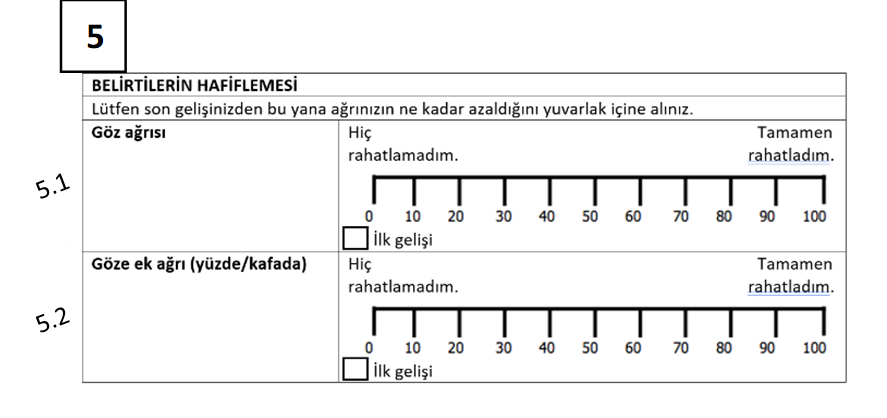

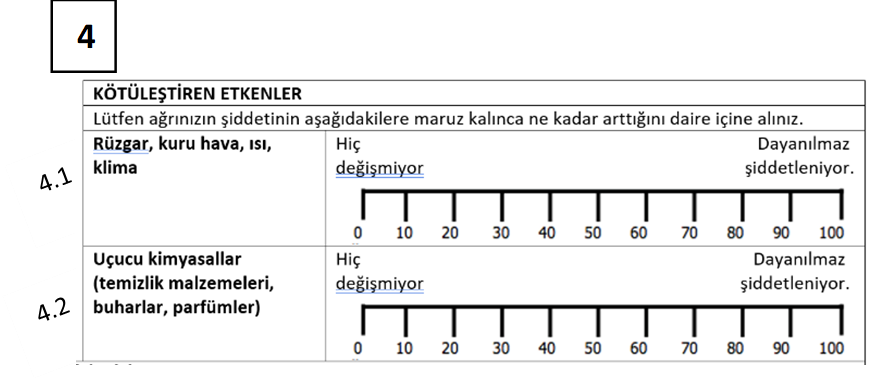

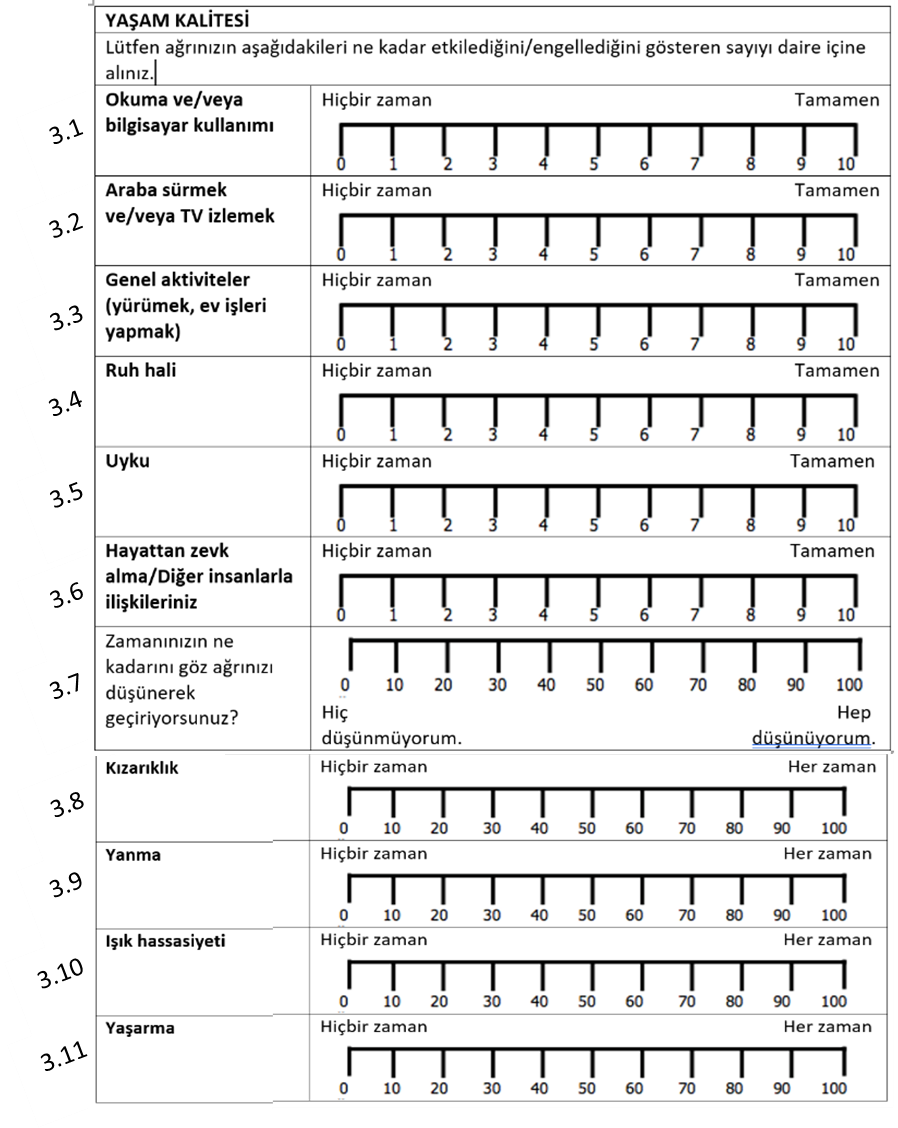

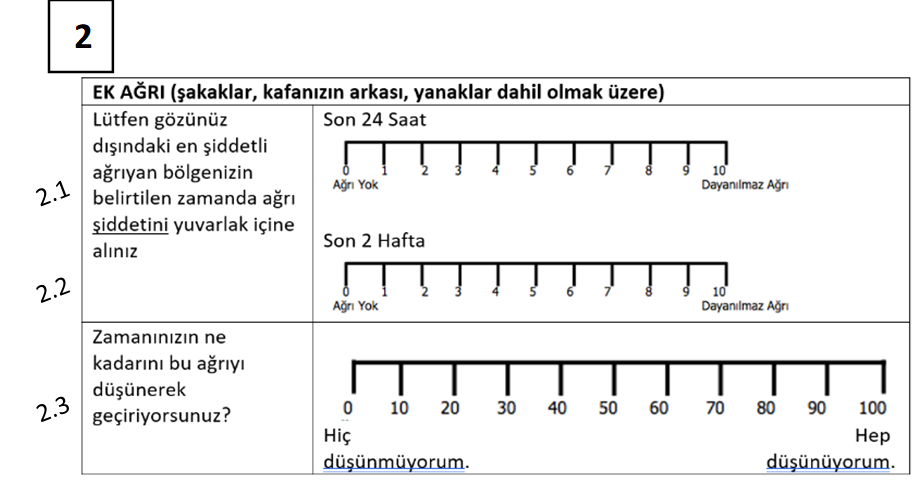

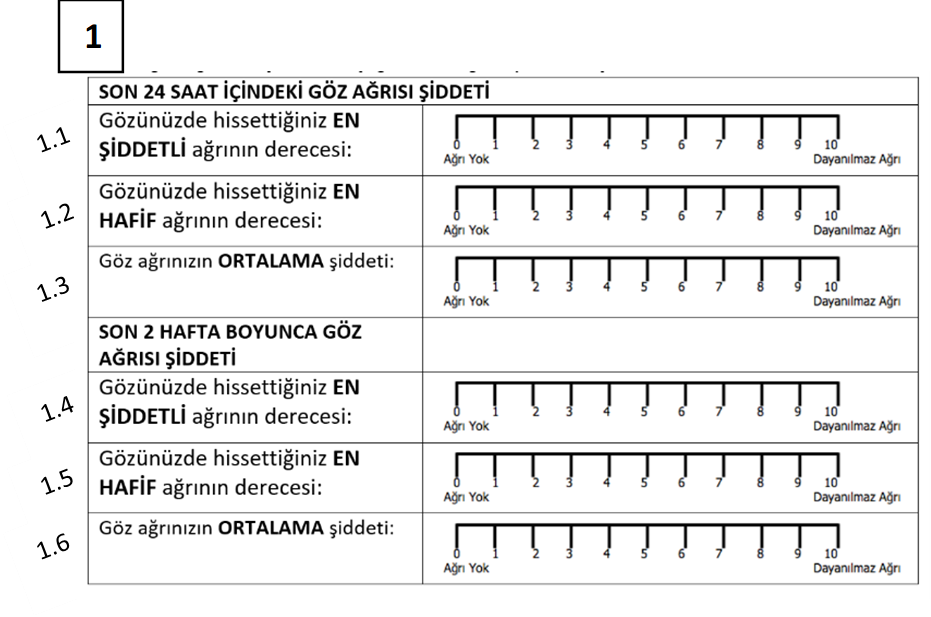


**3**


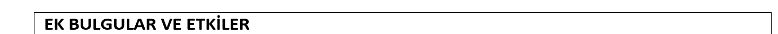

Supplement: Supplementary Materials — The final version of our adaptation of OPAS is available as a supplementary file. [file 3116913.f1.docx]
